# A modified Delphi approach to develop a trial protocol for antibiotic de-escalation in patients with suspected sepsis

**DOI:** 10.1017/ash.2021.205

**Published:** 2021-11-08

**Authors:** Michael E. Yarrington, Rebekah W. Moehring, Michael Z. David, Keith W. Hamilton, Michael Klompas, Chanu Rhee, Kevin Hsueh, Elizabeth Dodds Ashley, Ronda L. Sinkowitz-Cochran, Matthew Ryan, Deverick J. Anderson, for the DETOURS Expert Panel of the CDC Prevention Epicenters Program

**Affiliations:** 1 Duke Center for Antimicrobial Stewardship and Infection Prevention, Durham, North Carolina; 2 Division of Infectious Diseases, University of Pennsylvania, Philadelphia, Pennsylvania; 3 Department of Population Medicine, Harvard Medical School/Harvard Pilgrim Health Care Institute, Boston, Massachusetts; 4 Division of Infectious Diseases, Washington University School of Medicine, St Louis, Missouri; 5 Duke Center for Healthcare Safety and Quality, Durham, North Carolina; 6 Division of Healthcare Quality Promotion, Centers for Disease Control and Prevention, Atlanta, Georgia

**Keywords:** modified Delphi, antimicrobial stewardship, Sepsis, patient safety, protocol development

## Abstract

**Background:**

Early administration of antibiotics in sepsis is associated with improved patient outcomes, but safe and generalizable approaches to de-escalate or discontinue antibiotics after suspected sepsis events are unknown.

**Methods:**

We used a modified Delphi approach to identify safety criteria for an opt-out protocol to guide de-escalation or discontinuation of antibiotic therapy after 72 hours in non-ICU patients with suspected sepsis. An expert panel with expertise in antimicrobial stewardship and hospital epidemiology rated 48 unique criteria across 3 electronic survey rating tools. Criteria were rated primarily based on their impact on patient safety and feasibility for extraction from electronic health record review. The 48 unique criteria were rated by anonymous electronic survey tools, and the results were fed back to the expert panel participants. Consensus was achieved to either retain or remove each criterion.

**Results:**

After 3 rounds, 22 unique criteria remained as part of the opt-out safety checklist. These criteria included high-risk comorbidities, signs of severe illness, lack of cultures during sepsis work-up or antibiotic use prior to blood cultures, or ongoing signs and symptoms of infection.

**Conclusions:**

The modified Delphi approach is a useful method to achieve expert-level consensus in the absence of evidence suifficient to provide validated guidance. The Delphi approach allowed for flexibility in development of an opt-out trial protocol for sepsis antibiotic de-escalation. The utility of this protocol should be evaluated in a randomized controlled trial.

Early administration of antibiotics in sepsis is associated with reduced mortality.^1^ The Centers for Medicare and Medicaid Services (CMS) amended its Core Measures program in 2015 to mandate a National Quality Forum-endorsed quality measure (SEP-1) that required early recognition and bundled management of sepsis.^2^ The all-or-nothing sepsis bundle was based on elements from the Surviving Sepsis Campaign (SSC), which strengthened recommendations for sepsis management in 2016 to include broad-spectrum antibiotics administration within 1 hour of sepsis recognition.^3,4^ The strict time-oriented approach was criticized for the potential unnecessary or overly broad administration of antimicrobials among patients who ultimately did not have a diagnosis of infection or sepsis.^5–8^ In one study, ∼40% of patients with suspected sepsis were adjudicated to lack infection or represent “possible” sepsis.^9^ Although the SSC guidelines promote thoughtful, daily assessments for antibiotic de-escalation, it does not contain a de-escalation or discontinuation measure to avoid the unintended consequences of excess antibiotics. Standardized processes to encourage antibiotic de-escalation or discontinuation (hereafter assumed to be a subset of ‘de-escalation’) after suspected sepsis events need further development.

Antibiotic stewardship programs (ASPs) have experience performing antibiotic de-escalation reviews and protocolized interventions. For example, intravenous to oral conversion protocols performed by clinical pharmacists utilize standard eligibility criteria and provide a recommendation, while allowing the treating physician to decline the recommendation if appropriate.^10–13^ Antibiotic decision making is a highly nuanced thought process, requiring assessment and reassessment of multiple patient-specific clinical factors. Safety becomes a key focus when engaging clinicians in such complex decisions. Protocols that allow clinicians to “opt-out” of a process (ie, clinicians retain autonomy to accept for forego the ‘intervention’) provide a distinct advantage in gaining acceptance, especially when objective data elements in the medical record may not fully capture an individual clinical scenario. Opt-out protocols have been successfully used to improve HIV screening, to decrease unnecessary use of proton pump inhibitors, to avoid adverse outcomes among ventilated patients, and to prevent catheter-related infections.^14–17^ Patients started on broad-spectrum antibiotics because sepsis was initially suspected may benefit from an opt-out intervention to trigger antibiotic de-escalation or discontinuation.

In this study, we aimed to achieve consensus among members of an expert panel in defining objective high-risk exclusion (‘safety’) criteria, feasibly obtained during chart review, to identify patients unlikely to benefit from ongoing broad-spectrum antibiotics 72 hours after a suspected sepsis event. These criteria would then be used to develop an opt-out trial protocol to engage clinicians in discussion of antibiotic de-escalation for low-risk patients with suspected sepsis.

## Methods

We performed a modified Delphi, expert-consensus-building process to identify safety criteria for an opt-out protocol to guide de-escalation of antibiotic therapy among qualifying non-ICU adult patients with suspected sepsis. The modified-Delphi process included convening the expert panel, literature review and compilation of a list of candidate criteria, and 3 rounds of iterative discussion and electronic surveys to rate and then refine candidate criteria for the protocol.^18^ Expert panel discussions were held via web-based teleconferences. Participants completed 3 electronic surveys during calendar year 2017. The study was deemed exempt from review by the institutional review board.

### Convening the expert panel

Expert panelists were voluntary participants identified by the study team and CDC partners across multiple institutions in the United States by their experience in critical care, antimicrobial stewardship, infection prevention, and interests in sepsis care and research. Invited panelists were from prospective trial sites, which included 5 tertiary-care hospitals in the CDC Prevention Epicenters Program and 3 community hospital partners, in addition to CDC experts. Panelists were selected to produce a range of experience in both academic and community hospital settings, including pharmacists and physicians. The panel was moderated through the modified Delphi process by an expert in behavioral science (R.L.S.).

### Literature review and compilation of candidate safety criteria

The literature review to identify candidate safety criteria included a PubMed search of articles from the preceding 20 years using the the terms ‘sepsis’ and ‘septicemia.’ Articles were further narrowed to identify observational and experimental studies that focused on treatment of sepsis. The study team reviewed candidate articles with the expert panel for additional recommended studies. Candidate criteria from the following publication types were considered: (1) inclusion and exclusion criteria in sepsis treatment trials, (2) criteria used for antibiotic de-escalation and discontinuation in prospective trials of sepsis therapies, and (3) objective criteria used to define clinical response for patients presenting with sepsis in interventional and observational studies. The candidate criteria were reviewed internally by investigators and were categorized into 6 groups: study inclusion criteria, antibiotic exposures, comorbidities, laboratory data, signs and symptoms of infection, and severity of illness. The parameters were presented to the expert panel across 3 rounds with anonymous, electronic surveys. The expert panel was given the opportunity to add to the list of candidate parameters with each discussion.

### Electronic survey development

The list of parameters was compiled into electronic survey rating tools using Qualtrics survey software (Seattle, WA). The first electronic survey presented panelists with all criteria from the literature review. Panelists were asked to assign each criterion as “retain” (keep as is or modify) or “remove” (no longer consider for the protocol) according to their belief that the criterion would assist in identifying patients eligible for antibiotic de-escalation. Parameters with consensus (>66.6% agreement on either “retain” or “remove”) were presented during panel discussion for confirmation of results. Discussions then focused on addressing and voicing the rationales of panel members regarding ratings on criteria that did not achieve consensus. Parameters that were retained, modified, or did not achieve consensus continued to the next round.

The second and third electronic surveys used a standardized case-scenario format to present each criterion in clinical context. In the second survey, panelists were asked to answer a yes-or-no question in response to each clinical vignette that showed resolution of sepsis symptoms plus the criterion to be judged: “Should antibiotics be stopped in the patient on day 3 after suspected sepsis?” Because panelists had the opportunity in the first round to discuss rationales for criteria that did not achieve consensus, the minimum required concordance for retention or removal of criteria in round 2 was increased to 80%. Criteria that were below 90% but above 80% concordant were discussed with the expert panel for clarification and/or modification. Criteria that scored ≥90% agreement to stop antibiotics were removed from the pool without further discussion.

**Fig. 1.**
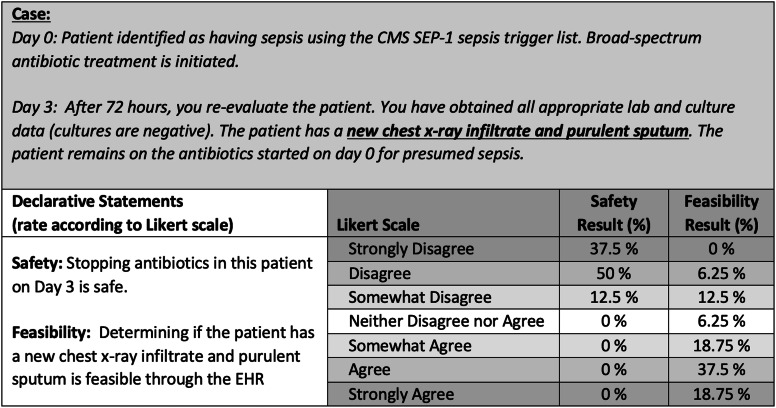
Case-scenario and safety and feasibility declarative statements example. Note. Each criterion was discussed during round 3 due to the survey results indicating <80% agreement in the feasibility of this parameter being identified by chart review. The expert panel agreed to modify and combine criteria to exclude patients with new chest radiograph infiltrate with or without purulent sputum.

The third electronic survey asked panelists to assess each criterion for feasibility and safety. Panelists were asked to review a case scenario and rate their agreement on a 7-point Likert scale for 2 declarative statements: “Stopping antibiotics on day 3 is safe” and “Determining this patient has (criterion) is feasible” (Fig. 1). Ratings were dichotomized into binary outcomes, and percent concordance for agree (‘somewhat agree’ through ‘strongly agree’) or disagree (‘strongly disagree’ through ‘neither’) were evaluated. Each criterion was evaluated for removal during panel discussion if the panel rated with >80% concordance that discontinuing antibiotics was safe or <80% concordance that the criteria could be feasibly identified by chart review. Criteria retained through 3 rounds and discussions were considered the final consensus criteria. Final criteria were included in a draft of the opt-out protocol safety-check screening criteria and were presented for review by the expert panel during the last panel meeting. Feedback, comments, and final suggestions were incorporated into the De-escalating Empiric Therapy: Opting Out of Rx for Suspected Sepsis, or “DETOURS,” trial protocol.

## Results

**Fig. 2.**
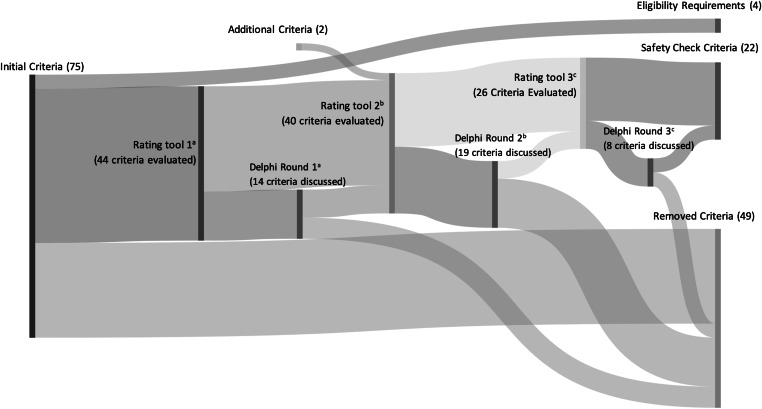
DETOURS panel criteria decisions flow diagram. (a) Round 1 had 19 survey responses and 13 panel members present for discussion. (b) Round 2 had 17 survey responses and 14 panel members present for discussion. (c) Round 3 had 17 survey responses and 11 panel members present for discussion. Note. Sankey diagram demonstrates criteria discussion, removal, and addition during each round of the Modified Delphi process. Detailed information on specific criteria is provided in Supplementary Table 1. DETOURS, De-escalating Empiric Therapy: Opting Out of Rx for Suspected Sepsis.

The expert panel included 23 members across 4 CDC Prevention Epicenters and 8 healthcare institutions. All members were retained through the conclusion of the Delphi process. Each survey had at least 17 respondents, and panel discussions included at least 11 members. Community hospitals, academic centers and CDC members were represented during each discussion. The literature review yielded 34 articles from which an initial list of 75 candidate criteria were reviewed (Supplementary Table 1). The candidate criteria were deduplicated to 48 unique criteria grouped into 6 categories: inclusion criteria, antibiotics, comorbidities, laboratory data, signs and symptoms, and disease severity (Fig. 2 and Supplementary Table 2). The 4 criteria identified as eligibility requirements for the proposed protocol were not included in the expert panel Delphi process: patient not located in intensive care unit, patient located on an adult medical or surgical unit, antibiotics initiated for suspected sepsis, and patient remained on any broad-spectrum antibiotics at 72 hours after the sepsis event.

### Round 1

The remaining 44 parameters were included in the first electronic survey in which panelists rated to retain or remove each criterion. Also, 14 parameters (32%) had a high degree of concordance (>66% of respondents) to either retain or remove. The expert panel discussion focused primarily on the criteria with a remove consensus; criteria with retain concordance would move on to round 2. Of the 10 criteria discussed, 6 were confirmed for removal and 4 parameters were retained. Criteria that the expert panel readily agreed to remove from consideration for the opt-out protocol in round 1 related primarily to patient demographics or comorbidities. For example, the criteria “patients from a nursing home” or “patients with a hospitalization in the prior 90 days” were deemed too restrictive and would limit eligibility for patients that would otherwise benefit from antibiotic de-escalation. In contrast, criteria related to specific clinical scenarios produced varied responses among panel members. For example, the panel debated over the criterion “exclude if leukocytosis is present (white blood cell count > 11,000 per mm^3^).” Optimal parameters for a white blood cell count threshold, or whether recent white blood cell values should be considered, were less clear. The panel retained the leukocytosis criterion with modification for subsequent rounds. During round 1, the panel recommended the addition of 2 criteria for review in subsequent rounds: “exclude if antibiotics administered prior to blood culture” and “exclude if patient is on perioperative prophylaxis.”

### Round 2

In total, 40 parameters were presented via the round 2 survey in case-based scenarios. The panel was >90% concordant on the decision to stop antibiotics in 12 case scenarios, and these criteria were removed from the pool after brief review. Further discussion with the expert panel involved 4 criteria that were near concordance (80%–90%) in antibiotic decision making: “patient has bronchiectasis,” “patient has asplenia,” “patient is pregnant,” and “patient is expected to die within 48 hours.” The first 3 criteria were retained to rate safety and feasibility in round 3, and the last was removed. The panel estimated cases with impending mortality to have no significant safety concern if the clinician retained the ability to opt out. To further simplify the remaining pool, the panel discussed criteria that were similar and could be combined. For example, “patients on high-dose, long-term corticosteroids” was incorporated into “patients on immunosuppressive agents.”

### Round 3

**Fig. 3.**
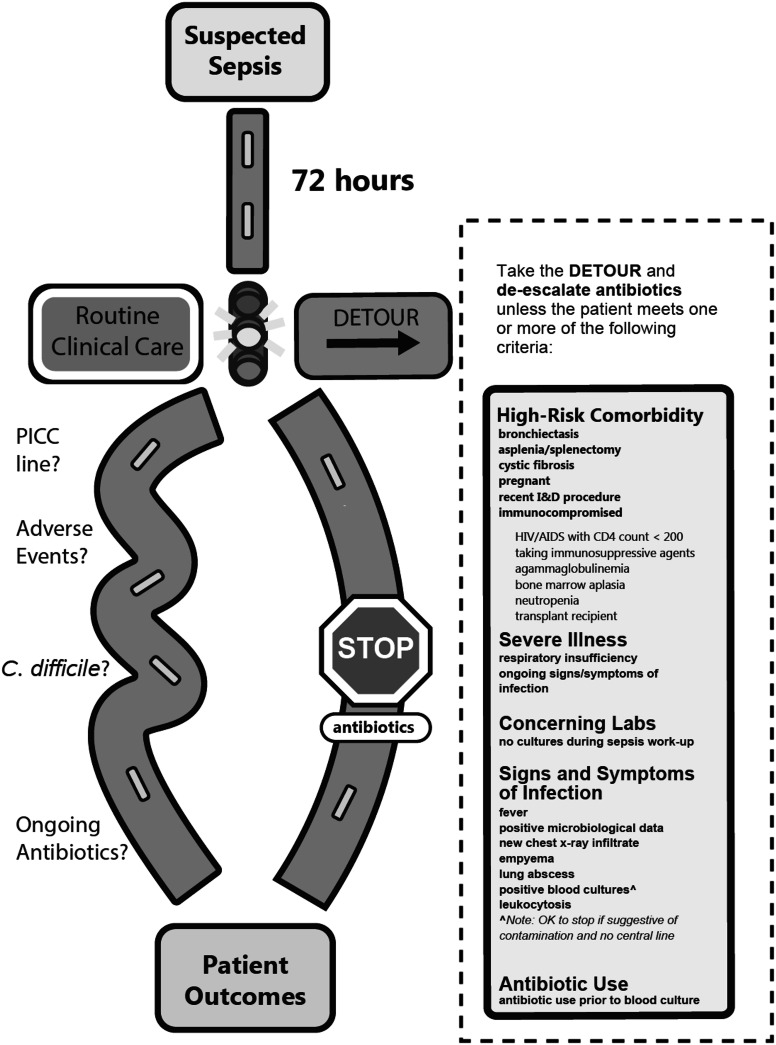
Flow chart with proposed criteria for DETOURS randomized, controlled trial. Note. DETOURS: De-escalating Empiric Therapy: Opting Out of Rx for Suspected Sepsis. Hashed box indicates the Opt-Out criteria sekected through the modified Delphi process.

The third survey included 26 criteria to evaluate feasibility and safety. In total, the expert panel discussed 5 parameters that scored <80% for feasibility or >80% for safety of stopping antibiotics. All criteria were retained; however, the panel further simplified criteria to increase feasibility for chart extraction. For example, 3 criteria that related to chest radiograph infiltrates plus additional findings were encompassed into the single parameter of “new chest x-ray infiltrate.” The remaining 22 criteria were placed into a draft for the opt-out protocol safety checklist. The protocol was reviewed with the expert panel with a mock-up graphic to obtain final comments (Fig. 3).

## Discussion

The primary finding of this protocol development program was that a multidisciplinary panel of experts in antimicrobial stewardship, critical care, and infectious diseases can achieve consensus through a modified Delphi approach to identify safety criteria for an opt-out protocol for antibiotic de-escalation in patients with suspected sepsis. Although previous studies have used a modified Delphi approach to identify hospital ASP structure and ASP metrics, to our knowledge, this is the first use of a modified Delphi approach to inform development of a randomized, controlled study trial protocol in antimicrobial stewardship.^19,20^

The Delphi approach is an important tool in the setting of incomplete knowledge, as it can synthesize the cumulative experience of an array of subject-matter experts. Antimicrobial de-escalation, although broadly recommended as a strategy in clinical guidelines, has limited evidence for safety in patients with sepsis, including few randomized controlled trials, and thus relies heavily on subject matter expertise.^21–23^ The modified Delphi approach allowed multiple participants with diverse expertise to discuss complex patient-care scenarios and achieve consensus on a set of safety criteria. The case-based format and anonymous survey rating tools were effective at highlighting different approaches to patient care among the DETOURS panel that result from a lack of standardized guidelines. At completion of the Delphi process, the DETOURS panel understood and agreed with the rationale behind each criterion in the final opt-out protocol. Furthermore, the panel process promoted familiarity with the study protocol among stakeholders at institutions intended to be part of the randomized, controlled trial.

The DETOURS panel discussed 2 key factors when reviewing criteria for antibiotic de-escalation: patient safety and feasibility. Discussions of each criterion focused primarily on patient safety as well as identifying scenarios where benefit would be gained by review for de-escalation. The proposed intervention required clinician engagement in evaluating patients for antibiotic discontinuation or de-escalation. Panel members were inclined to identify criteria that would promote safe recommendations from the local stewardship team and were feasible for electronic chart abstraction. Any criteria that required detailed, bedside knowledge of the patient were deemed impractical. Detailed criteria that remained at the end of the Delphi process are provided in the Supplementary Material.

Development of an opt-out protocol, in which clinicians reserve final decision-making power, allow greater flexibility in interpretation of each criterion or clinical scenario because the opt-out mechanism maintains clinician autonomy as a back-stop for safety. For example, the ‘Wake Up and Breath Collaborative’ demonstrated the utility and safety of an opt-out protocol for evaluating the appropriateness of spontaneous breathing trials (SBTs) and spontaneous awakening trails (SATs) in ventilated patients on a daily basis. The increased monitoring was associated with lower rates of ventilator-associated events, yet the decision to proceed safely with SBTs and SATs remained with clinicians.^16^ Similarly, the DETOURS panel did not need to achieve perfection in selection of the opt-out protocol safety criteria, knowing that clinical judgments could overrule the criteria obtained from chart review. For example, the criterion to exclude patients with expected mortality within 48 hours was initially included for evaluation by the panel due to patient safety concerns. However, end-of-life care also provides opportunities for antibiotic de-escalation.^24^ In the opt-out protocol, clinicians would retain the ability to continue antimicrobials in cases of patient safety or individual patient/family goals. Thus, the panel removed this exclusion, allowing end-of-life scenarios to be evaluated for de-escalation opportunities.

Our study using the modified Delphi approach had limitations. First, the expert panel was large and was composed of members across multiple centers in the United States; thus, not every member could participate on every task, and time was limited in each teleconference discussion. Due to the panel composition, generalizablility outside the United States was also limited. However, the structure allowed for a diversity of opinions, and all members did have opportunity to participate in written surveys. Second, the focus on patient safety may have led to overly narrow criteria and more “missed opportunities” for de-escalation. Finally, the criteria evaluated by the expert panel did not capture some specific patient scenarios (ie, asymptomatic bacteriuria, endocarditis, osteomyelitis, or medical antibiotic prophylaxis) that may or may not appropriately trigger a review for antibiotic de-escalation.

The modified Delphi approach and expert panel review process led to the development of safety criteria for an opt-out protocol to guide antibiotic cessation or de-escalation after initiation of antibiotics for suspected sepsis. The efficacy of this protocol as a tool to improve antibiotic use will require prospective evaluation in a randomized controlled trial.

## DETOURS Expert Panel

Deverick Anderson MD, MPH, Elizabeth Dodds-Ashley PharmD, Rebekah Moehring MD, MPH, Cara O’Brien MD, Christina Sarubbi MBA, PharmD, Rebekah Wrenn PharmD, Duke University; Charles Callahan MD, Cleveland Clinic Indian River Hospital; Christine Zurawski MD, Brett Sandifer MD, Piedmont Healthcare; Kevin Hsueh MD, Tiffany Osborn MD, Robert Martin MD, Holly Beiter MSN, MBA, Washington University; Michael Klompas MD, MPH, Chanu Rhee MD, MPH, Harvard University; Michael David MD, Keith Hamilton MD, Mark Mikkelesen MD, Craig Umscheid MD, Bill Schweickert MD, University of Pennsylvania; Tony Fiore MD, John Jernigan MD, Sujan Reddy MD, The Centers for Disease Control and Prevention; Marcos Restrepo MD, University of Texas Health Science Center at San Antonio.

## Supplementary material

For supplementary material accompanying this paper visit https://doi.org/10.1017/ash.2021.205. click here to view supplementary material
