# Supplementary material for: A modified Delphi approach to develop a trial protocol for antibiotic de-escalation in patients with suspected sepsis
Source: Antimicrob Steward Healthc Epidemiol. 2021 Nov 8;1(1):e44. doi: 10.1017/ash.2021.205 (PMC9495406; doi:10.1017/ash.2021.205)
Supplement: Supplementary file 1 [file S2732494X21002059sup001.docx]

Supplementary Table 1: Journal Articles Used for Criteria Selection

| **article** | **authors** | **year** | **title** | **journal** |
| --- | --- | --- | --- | --- |
| 1 | Chastre et al. | 2003 | Comparison of 8 vs 15 days of antibiotic therapy for ventilator-associated pneumonia in adults: a randomized trial | JAMA |
| 2 | Daneman et al. | 2015 | Bacteremia Antibiotic Length Actually Needed for Clinical Effectiveness (BALANCE): study protocol for a pilot randomized controlled trial | Trials |
| 3 | Morel et al. | 2010 | De-escalation as part of a global strategy of empiric antibiotherapy management. A retrospective study in a medico-surgical intensive care unit | Critical Care |
| 4 | Garnacho-Montero et al. | 2014 | De-escalation of empirical therapy is associated with lower mortality in patients with severe sepsis and septic shock | Intensive Care Med |
| 5 | Moussaoui et al. | 2006 | Effectiveness of discontinuing antibiotic treatment after three days versus eight days in mild to moderate-severe community acquired pneumonia: randomised, double blind study | BMJ |
| 6 | Ross et al. | 2016 | Safety of Automatic End Dates for Antimicrobial Orders to Facilitate Stewardship | ICHE |
| 7 | Rubin, Buehler and Halpern | 2016 | States Worse than Death Among Hospitalized Paitents with Serious Illnesses | JAMA Internal Med |
| 8 | Lavrentieva et al. | 2017 | Determinants of Outcome in Burn ICU Patients with Septic Shock | Journal of Burn Care and Research |
| 9 | Guo et al. | 2016 | De-escalation of empiric antibiotics in patients with severe sepsis or septic shock: A meta-analysis | Heart & Lung |
| 10 | Leone et al. | 2014 | De-escalation versus continuation of empirical antimicrobial treatment in severe sepsis: a multicenter non-blinded randomized noninferiority trial | Intensive Care Med |
| 11 | Ferrer er al. | 2014 | Empiric antibiotic treatment reduces mortality in severe sepsis and septic shock from the first hour: results from a guideline-based performance improvement program. | Critical Care Medicine |
| 12 | Paskovaty et al. | 2015 | Antimicrobial de-escalation in septic cancer patients: is it safe to back down? | Intensive Care Med |
| 13 | Mokart et al. | 2014 | De-escalation of antimicrobial treatment in neutropenic patients with severe sepsis: results from an observational study | Intensive Care Med |
| 14 | Murray et al. | 2014 | A multidisciplinary intervention to reduce antibiotic duration in lower respiratory tract infections. | Journal of Antimicrobial Therapy |
| 15 | Connor et al. | 2007 | Impact of Automatic Orders to Discontinue Vancomycin Therapy on Vancomycin Use in an Antimicrobial Stewardship Program | ICHE |
| 16 | Do et al. | 2012 | Audit of antibiotic duration of therapy, appropriateness and outcome in patients with nosocomial pneumonia following the removal of an automatic stop-date policy | European journal of clinical microbiology & infectious diseases |
| 17 | Kumar, A. | 2009 | Optimizing Antimicrobial Therapy in Sepsis and Septic Shock | Critical Care Clinics |
| 18 | Uranga, A. | 2016 | Duration of Antibiotic Treatment in Community-Acquired Pneumonia: A Multicenter Randomized Clinical Trial. | JAMA Internal Med |
| 19 | Bloos et al. | 2016 | Effect of Sodium Selenite Administration and Procalcitonin-Guided Therapy on Mortality in Patients With Severe Sepsis or Septic Shock: A Randomized Clinical Trial. | JAMA Internal Med |
| 20 | Daneman et al. | 2016 | Duration of Antimicrobial Treatment for Bacteremia in Canadian Critically Ill Patients. | Critical Care Medicine |
| 21 | Yamana et al. | 2016 | De-escalation versus continuation of empirical antimicrobial therapy in community-acquired pneumonia. | Journal of Infection |
| 22 | De Bus et al. | 2016 | Impact of de-escalation of beta-lactam antibiotics on the emergence of antibiotic resistance in ICU patients: a retrospective observational study. | Intensive Care Med |
| 23 | Madaras-Kelly et al. | 2015 | Antimicrobial de-escalation of treatment for healthcare-associated pneumonia within the Veterans Healthcare Administration. | Journal of Antimicrobial Chemotherapy |
| 24 | Salahuddin et al. | 2016 | Determinants of Deescalation Failure in Critically Ill Patients with Sepsis: A Prospective Cohort Study | Critical Care Research and Practice |
| 25 | Albrich and Harbarth | 2015 | Pros and cons of using biomarkers versus clinical decisions in start and stop decisions for antibiotics in the critical care setting. | Intensive Care Med |
| 26 | Sawyer et al. | 2015 | Trial of short-course antimicrobial therapy for intraabdominal infection. | NEJM |
| 27 | Nakamura et al. | 2015 | Potential use of procalcitonin as biomarker for bacterial sepsis in patients with or without acute kidney injury. | Journal of Infection and Chemotherapy |
| 28 | Edgeworth et al. | 2014 | Long-term adherence to a 5 day antibiotic course guideline for treatment of intensive care unit (ICU)-associated Gram-negative infections. | Journal of Antimicrobial Chemotherapy |
| 29 | Paul et al. | 2016 | Antibiotic de-escalation for bloodstream infections and pneumonia: systematic review and meta-analysis. | Clinical Microbiology and Infection |
| 30 | de Jong et al. | 2016 | Efficacy and safety of procalcitonin guidance in reducing the duration of antibiotic treatment in critically ill patients: a randomised, controlled, open-label trial. | The Lancet Infectious Diseases |
| 31 | Lee et al. | 2015 | Impact of antimicrobial strategies on clinical outcomes of adults with septic shock and community-onset Enterobacteriaceae bacteremia: de-escalation is beneficial. | Diagnostic Microbiology and Infectious Disease |
| 32 | Hayashi, Peterson | 2011 | Strategies for Reduction in Duration of Anitbiotic Use in Hospitalized Patients | Clinical Infectious Diseases |
| 33 | Klompas et al. | 2017 | Ultra-Short-Course Antibiotics for Patients With Suspected Ventilator-Associated Pneumonia but Minimal and Stable Ventilator Settings | Clinical Infectious Diseases |
| 34 | Micek et al. | 2004 | A Randomized Controlled Trial of an Antibiotic Discontinuation Policy for Clinically Suspected Ventilator- Associated Pneumonia | Clinical Investigations in Critical Care |

Supplementary Table 2. Criteria Evaluated during DETOURS Modified Delphi Review

| **Rating Tool 1 (n=44)** | **Rating Tool 2 (n=40)** | **Rating Tool 3 (n=26)** | **Final Criteria (n=22)** |
| --- | --- | --- | --- |
| Antibiotics initiated for suspected sepsis |  |  | Antibiotics initiated for sepsis |
| Not in ICU |  |  | Not in ICU |
| On adult medical/surgical acute care ward | |  | On adult medical/surgical acute care ward |
| Still on antibiotics at 72 hours |  |  | Still on antibiotics at 72 hours |
|  | **Antibiotics** |  |  |
| On prophylactic antibiotics |  |  |  |
| Antibiotics for other study/protocol |  |  |  |
|  | (Antibiotics prior to blood cultures) |  | Antibiotics prior to blood cultures |
|  | (Peri-operative prophylaxis) |  |  |
|  | **Co-Morbidities** |  |  |
| Bronchiectasis |  |  | Bronchiectasis |
| Agammaglobulinemia |  |  | Agammaglobulinemia |
| Asplenia |  |  | Asplenia |
| Bone marrow aplasia |  |  | Bone marrow aplasia |
| Cystic fibrosis |  |  | Cystic fibrosis |
| Neutropenia |  |  | Neutropenia |
| Pregnant |  |  | Pregnant |
| Taking immunosuppressive agent |  |  | Taking immunosuppressive agent |
| Transplant recipient |  |  | Transplant recipient |
| HIV with CD4<200 |  |  | HIV with CD4<200 |
| Recent procedure - I&D |  |  | Recent procedure - I&D |
| Recent procedure - general anesthesia |  |  |  |
| HIV infection |  |  |  |
| Breastfeeding |  |  |  |
| ESRD requiring HD |  |  |  |
| Hospice |  |  |  |
| Prolonged stay in ICU (>96 hours) |  |  |  |
| Corticosteroids |  |  |  |
| Prisoner |  |  |  |
| Admitted from nursing home |  |  |  |
| Prosthetic valve |  |  |  |
| Recent hospitalization (last 90 days) |  |  |  |
|  | **Laboratory Data** |  |  |
| Cultures not obtained for sepsis work-up |  |  | Cultures not obtained for sepsis work-up |
| PCT suggestive of bacterial infection |  |  |  |
|  | **Signs/Symptoms** |  |  |
| Empyema |  |  | Empyema |
| Fever |  |  | Fever |
| Leukocytosis |  |  | Leukocytosis |
| Lung abscess |  |  | Lung abscess |
| Blood culture suggestive of contamination |  |  |  |
| Positive blood cultures |  |  | Positive blood cultures not suggestive of contamination |
| Positive micro data |  |  | Positive micro data |
| New CXR infiltrate (but no other s/s of infection) |  |  | New CXR infiltrate |
| New CXR infiltrate + fever |  |  |  |
| New CXR infiltrate + positive sputum cx |  |  |  |
| New CXR infiltrate + purulent sputum |  |  |  |
|  | **Severity** |  |  |
| Respiratory insufficiency |  |  | Respiratory insufficiency |
| Non-bacterial infection |  |  | Non-bacterial infection + s/s of infection |
| Admitted with cardiopulmonary arrest |  |  |  |
| Expected death (<48 hours) |  |  |  |
| Expected withdrawal of care |  |  |  |
| Hospital duration >5 days |  |  |  |
| Discharged from ICU within 72 hours |  |  |  |
| Green indicates a parameter was retained in round discussion after rating tool was disseminated among the panel members.  Yellow indicates a parameter was modified during round discussion  Red indicates parameter was removed  Boxed fields indicate the parameter was discussed during among the expert panel teleconference  Parenthesis indicate parameter was added after recommendation from expert panel discussion | | | |
